# Efficacy and Safety of MED-01 Probiotics on Vaginal Health: A 12-Week, Multicenter, Randomized, Double-Blind, Placebo-Controlled Clinical Trial

**DOI:** 10.3390/nu15020331

**Published:** 2023-01-09

**Authors:** Sung-Ho Park, Eun Sil Lee, Sung Taek Park, Soo Young Jeong, Yeoul Yun, YongGyeong Kim, Yulah Jeong, Chang-Ho Kang, Hyun Jin Choi

**Affiliations:** 1 Hallym University Kangnam Sacred Heart Hospital, 665 Siheung-daero, Yeongdeungpo-gu, Seoul 07442, Republic of Korea; 2 Soonchunhyang University Seoul Hospital, 59 Daesagwan-ro, Yongsan-gu, Seoul 04401, Republic of Korea; 3 Mediogen, Co., Ltd., Biovalley 1-ro, Jecheon-si 27159, Chungcheongbuk-do, Republic of Korea; 4 Department of Obstetrics and Gynecology, College of Medicine Chung-Ang University, Chung-Ang University Gwangmyeong Hospital, 501 Iljik-dong, Gwangmyeong-si 14353, Gyeonggi-do, Republic of Korea

**Keywords:** bacterial vaginosis, probiotics, MED-01

## Abstract

Bacterial vaginosis (BV) is the most common disease in women of childbearing age and is caused by the growth of abnormal microbiota in the vagina. Probiotic consumption can be an effective alternative treatment to preserve or improve vaginal health. In the present study, MED-01, a complex of five strains of probiotic candidates isolated from the vagina of Korean women, was used. This study was designed as a 12-week, randomized, multicenter, double-blind, placebo-controlled clinical trial to evaluate the efficacy and safety of MED-01 on vaginal health. A total of 101 reproductive-aged women with a Nugent score of 4–6 took MED-01 (5.0 × 10^9^ CFU) or a placebo once a day, and 76 participants completed the procedure. MED-01 significantly reduced the Nugent score compared with the placebo. Quantitative PCR analysis confirmed that *Lactobacillus plantarum* was significantly increased in the vagina, whereas harmful bacteria such as *Mobiluncus* spp., *Gardnerella vaginalis,* and *Atopobium vaginae* were suppressed after 12 weeks of MED-01 ingestion. No adverse events to the test food supplements were observed in the participants. These results confirmed that MED-01 can be used as a probiotic for treating BV, as it improves the vaginal microbiota.

## 1. Introduction

Bacterial vaginosis (BV) is characterized by the depletion of lactic acid bacteria (LAB) and overgrowth of certain anaerobic and facultative bacteria such as *Gardnerella vaginalis* and *Atopobium vaginae*, leading to an imbalance in the vagina [1]. BV is associated with pelvic inflammatory diseases and gynecological illnesses such as late miscarriages, premature rupture of membranes, and preterm birth [2,3]. In addition, BV has been strongly linked to an increased risk of human immunodeficiency virus-1 transmission [4]. Half of the women treated for BV experience a recurrence within 12 months [5]. The high recurrence rate of BV results in repeated exposure to antibiotics and the emergence of drug-resistant bacteria [6]. Repeated treatment of BV leads to an imbalance in the vaginal environment and a high recurrence rate of vaginal infections. Therefore, alternative therapeutic methods are needed to restore the healthy vaginal microbiota.

Probiotics are now recognized as an alternative or additional treatment method for BV. The World Health Organization defines probiotics as “live microorganisms which, when administered in adequate amounts, confer a health benefit on the host” [7]. Lactobacilli, which includes *Lactobacillus crispatus*, *Lactobacillus iners*, etc., are the most abundant bacteria in the healthy vaginas of childbearing age women in Korea [8,9]. Lactobacilli play an important role in maintaining a healthy vaginal environment [10]. The vaginal ecosystem is balanced by various Lactobacilli producing several inhibitory compounds, such as hydrogen peroxide, lactic acid, and bacteriocins, which prevent the growth of pathogens as well as the adherence of pathogens to the vaginal epithelium [10,11,12]. Various studies have shown that probiotics relieve BV and reduce the rate of BV recurrence [11,13,14]. However, not all probiotics are effective against vaginitis [15,16]. Therefore, to use probiotics for vaginal health, it is necessary to determine the efficacy of a probiotic through an actual human application test.

MED-01 is a complex that includes five probiotic strains, *Ligilactobacillus salivarius* MG242, *Limosilactobacillus fermentum* MG901, *Lactiplantibacillus plantarum* MG989, *Lacticaseibacillus paracasei* MG4272, and *Lacticaseibacillus rhamnosus* MG4288, which were isolated from the vagina of Korean women. In our previous studies, these strains showed in vitro antibacterial effects on the causative bacteria of BV and relieved inflammation by inhibiting pathogen adhesion. Moreover, orally administered MED-01 reduces inflammatory cytokines and myeloperoxidase (MPO) activity and inhibits the viability and attachment of vaginal *G. vaginalis* in BV-induced mice [17,18]. However, the efficacy of MED-01 in humans remains uninvestigated.

Therefore, in the present study, a human application test was performed to evaluate the efficacy of MED-01 on vaginal health and safety. Its effects on vaginal health parameters (e.g., vaginal pH and Nugent score) and subjective symptoms such as vaginal discharge and burning sensation were also monitored.

## 2. Materials and Methods

### 2.1. Ethics

The present study was designed as a 12-week, randomized, multicenter, double-blind, placebo-controlled clinical trial with two parallel groups receiving either MED-01 or placebo capsules. The study was performed from January 2021 to April 2022 at Chung-Ang University Hospital, Hallym University Kangnam Sacred Heart Hospital, and Soonchunhyang University Hospital in Seoul, Korea. Prior to the implementation of the trial, the study protocol and consent form were reviewed and approved by the independent clinical trial review board (IRB) of each institution (IRB No. 2091-003-436, HKS 2020-09-005-001, and SCHUH 2021-04-017-002). The information about a clinical trial was registered at the Clinical Research Information Service, the Republic of Korea (accessed on 6 January 2023, https://cris.nih.go.kr/cris/search/detailSearch.do?search_lang=E&focus=reset_12&search_page=L&pageSize=10&page=undefined&seq=18347&status=5&seq_group=18347) and conducted in accordance with the ethical principles of the Declaration of Helsinki and Korea Good Clinical Practice.

### 2.2. Participants (Subjects)

All subjects (women of reproductive age; 19–50 years) participating in the screening visit were explained the purpose and protocol of this study and the foreseeable risks associated with the trial. Subjects who provided written informed consent to participate in the trial were screened for eligibility by collecting vaginal swabs, blood samples, and medical history and performing a physical examination. Subjects who met the inclusion criteria with a Nugent score of 4–6 were asked to visit within 14 days to enroll in the trial.

The exclusion criteria for the protocol were as follows: (1) undergoing treatment for severe cardiovascular system, immune system, respiratory system, gastrointestinal tract/liver and biliary system, kidney and urinary system, nervous system, musculoskeletal system, psychiatric, infectious disease, and malignant tumor, (2) vaginal or urinary tract infection, bleeding, or abnormality, (3) pregnant or planning pregnancy, (4) breast feeding, (5) using antibiotics, antibacterial agents, steroids, and immunosuppressants, (6) regularly using health functional foods, probiotics, and lactobacillus products, (7) experience of acupuncture treatment for women’s cleanser, vaginal cleanser, sedentary treatment, and vaginal health (within one week of screening visit), (8) uncontrolled hypertension (above 160 mmHg of systolic blood pressure or 100 mmHg of diastolic blood pressure), (9) unregulated diabetics (HbA1C > 6.5%), (10) abnormal creatinine, aspartate aminotransferase or alanine aminotransferase, thyroid stimulating hormone levels, (11) allergic to ingredients of this human-applied test food supplements, and (12) participated in another intervention clinical trial.

At the randomization visit (baseline), all subjects who met the inclusion/exclusion criteria were assigned to each group according to 1:1 randomization method. The number of subjects in each group was kept equal for balanced randomization between the intake groups. The randomization table was generated by sequentially applying permutations of random numbers (random numbers A and B) generated by the randomization program of the SAS^®^ system (Version 9.4; SAS Institute, Cary, NC, USA).

### 2.3. MED-01 and Placebo Capsules

The MED-01 capsules contained 5.0 × 10^9^ CFU probiotic strains with 1.0 × 10^9^ CFU of *L. salivarius* MG242, *L. fermentum* MG901, *L. plantarum* MG989, *L. paracasei* MG4272, and *L. rhamnosus* MG4288 each. In contrast, the placebo capsules contained only maltodextrin. The participants took 500 mg MED-01 or placebo capsules with water once daily for 12 weeks. To confirm the effects of the investigated food supplements, all participants visited the clinic at 6 and 12 weeks of the trial, and their anthropometric parameters were assessed.

### 2.4. Outcomes

#### 2.4.1. Nugent Score

The primary endpoint of MED-01 was a change in the Nugent score between baseline and 12 weeks after randomization. To reduce the multicenter error, all collected samples from the subjects at screening visits and 6 and 12 weeks were sent to an external centralized analysis institution (Korea Clinical Laboratory, Seoul, Korea) within 48 h. A trained professional prepared vaginal swabs and smears and stained the smears. Scoring was performed by a qualified microbiologist to increase the reliability of the test results. Gram staining of the vaginal smears was used to determine the Nugent score (Table 1) [19].

#### 2.4.2. Vaginal Conditions and Symptoms

Secondary efficacy of MED-01 was evaluated by changes in vaginal pH, vaginal microbiota, and symptom questionnaire assessments (vaginal discharge, odor, burning sensation, and dysuria). Vaginal pH was measured using pH test strips pH 3.8–5.4 (DF^®^; Guangzhou, China). The symptom questionnaire was given at visits 2, 3, and 4. For each question, “none (0 points)”, “weak (1 point)”, “moderate (2 points)”, “severe (3 points)”, and “very severe (4 points)” scales were used to determine the degree of symptoms experienced by the subject.

#### 2.4.3. Vaginal Microbiota

The vaginal swabs were collected to analyze vaginal microbiota from the participants at screening visits and 6 and 12 weeks. The sterile cotton swab was rolled across the mid-vaginal wall of each woman and stored at 2–8 °C until 7 days. Total DNA was extracted from vaginal swabs using the MagListo™ Genomic DNA Extraction Kit for ExiPrep^TM^ 96 Lite and the automated system ExiPrep^TM^ 96 Lite (Bioneer, Daejeon, Republic of Korea) according to the manufacturer protocols. The vaginal microbiota was detected using quantitative polymerase chain reaction (qPCR), which can measure the amplification products and cycles during the amplification reaction of the target DNA sequence in real-time. The analysis of vaginal microbiota was performed at the Korea Clinical Laboratory (Seoul, Republic of Korea). *G. vaginalis* was analyzed using AccuPower STI4C-Plex Real-Time PCR Kit (Bioneer, Daejeon, Republic of Korea) according to the manufacturer protocols. The primer sequences used for the analysis of other strains are shown in Supplementary Table S1. The PCR reaction was pre-cycling at 95 °C for 3 min, followed by 40 cycles of denaturation (95 °C, 10 s), annealing (56 °C, 20 s), and extension (72 °C, 20 s).

### 2.5. Safety Assessment

At visits 1 (week 0) and 4 (week 12), the safety of the investigated food supplements was evaluated by monitoring all adverse events (AEs), vital signs, blood pressure, pulse rate, body weight, urine analysis, hematological and blood chemistry tests (liver and renal function) of the subjects.

### 2.6. Statistical Analysis

Statistical analyses were conducted using SAS^®^ (Version 9.4; SAS Institute, Cary, NC, USA). To evaluate the effectiveness of MED-01, the per-protocol dataset (PP set) was used for the main analysis. The safety set for evaluating the safety of MED-01 included subjects who consumed the food supplement at least once after being randomly assigned in the human application test.

The significance of the differences in demographic characteristics at baseline was analyzed using the chi-square test, Fisher’s exact test, Wilcoxon rank sum test, or two-sample *t*-tests. The significance of the differences in the changes between the baseline and 12 weeks among groups was analyzed using a paired *t*-test. The differences in the changes from baseline to 6 and 12 weeks between the placebo and MED-01 groups were analyzed using a two-sample *t*-test or Wilcoxon rank sum test. Additionally, an analysis of covariance was conducted with the variables bidet or tampon use and amount of smoking and frequency of intercourse [20,21,22,23,24]. The data are presented as mean ± standard deviation (SD), and the significance of the differences was verified using a two-sided test at *p* < 0.05.

## 3. Results

### 3.1. Participants

**Figure 1:**
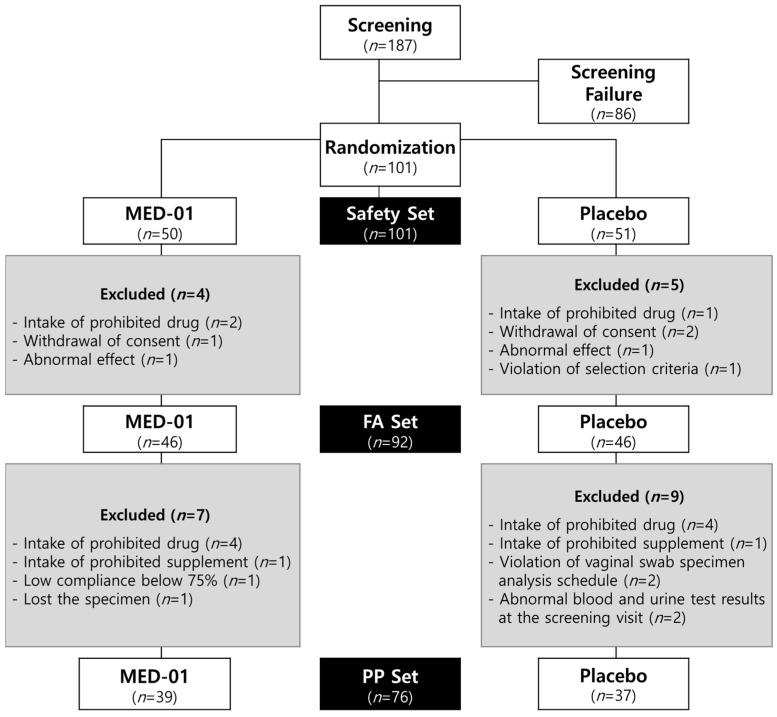
Flow diagram illustrating the selection of participants.

A total of 187 women were enrolled in the trial, of which 101 were randomized: 50 were assigned to the MED-01-intake group and 51 to the placebo-intake group (Figure 1). However, 9 out of the 101 subjects were excluded due to withdrawal of consent, use of prohibited drugs, unexpected AEs unrelated to MED-01 (gas/bloating, abdominal distension, nausea, constipation, and dyspepsia), or violation of the selection criteria. Finally, 46 subjects in the MED-01-intake group and 46 subjects in the placebo-intake group (FA set, *n* = 92) completed the trial.

However, seven and nine subjects meeting the exclusion criteria were excluded from the MED-01 and placebo groups in the FA set, respectively. Thus, the efficacy of MED-01 was finally evaluated in 39 and 37 subjects in the MED-01 and placebo groups, respectively, without significant violations affecting the test results (PP set, *n* = 76).

### 3.2. Baseline Characteristics of Participants

The demographic information of the two groups at the baseline is shown in Table 2. Demographic or lifestyle characteristics, including age, drinking, smoking, and frequency of exercising and showering, were not significantly different between the two groups.

In addition, the mean of adherence to the assigned supplement intake was 96.56 ± 10.55% and 96.53 ± 10.01% for the MED-01 and placebo groups, respectively, and no differences were observed between the two groups (*p* = 0.930 by Wilcoxon rank sum test).

### 3.3. Effect of MED-01 on the Nugent Score and Vaginal pH 

**Figure 2:**
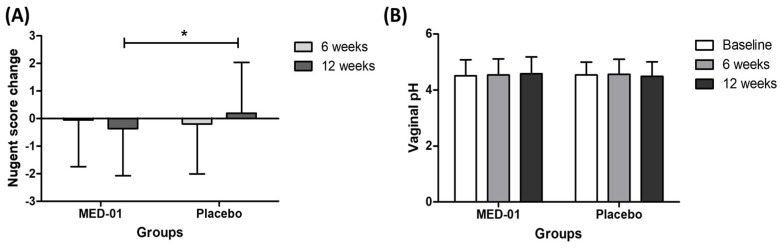
Effect of MED-01 on (**A**) the Nugent score and (**B**) vaginal pH from baseline to 12 weeks after randomization; Values are presented as mean ± SD; * *p* < 0.05 derived from an analysis of covariance (ANCOVA) between groups, MED-01 vs. placebo.

Changes in the Nugent score after 12 weeks of MED-01 intake were analyzed as a primary outcome. The Nugent score in the MED-01-intake group decreased by −0.36 ± 1.72, whereas it increased by 0.19 ± 1.85 in the placebo-intake group, showing a statistically significant difference between the two groups (*p* = 0.041, Figure 2A). However, during the trial period, the mean vaginal pH only varied between 4.49 and 4.58, and no statistically significant difference was observed between the two groups (Figure 2B).

Among the total subjects (Nugent score 4-6), 10 subjects (25.6%) in the MED-01 group improved to a normal score (0–3), which is twice that of 5 subjects (13.5%) in the placebo group. On the other hand, the number of subjects whose Nugent score rose to the BV stage was 6 (15.4%) and 8 (21.6%), respectively. However, there was no statistical significance between groups (Table 3).

### 3.4. Effect of MED-01 on Vaginal Symptoms

**Figure 3:**
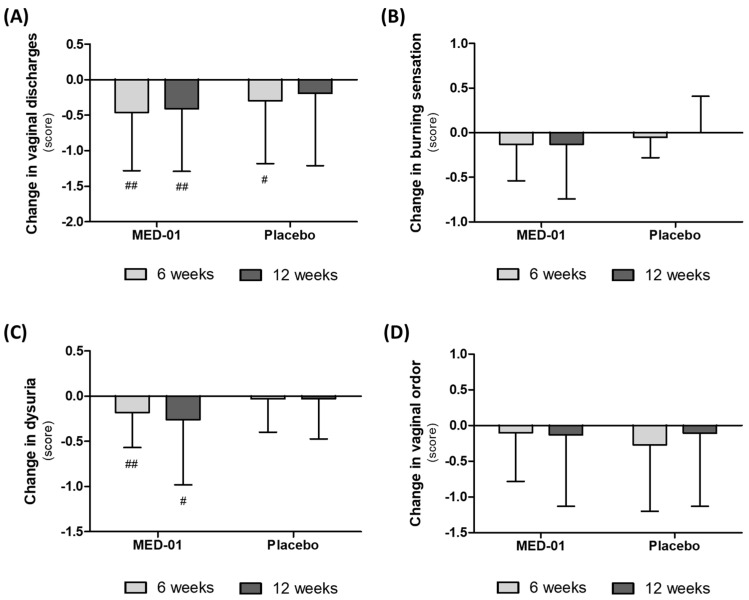
Effect of MED-01on the vaginal symptoms evaluated by the questionnaire evaluation from baseline to 12 weeks. Change in (**A**) vaginal discharges, (**B**) burning sensation, (**C**) dysuria, and (**D**) vaginal odor. Values are presented as the mean of score ± SD. # *p* < 0.05 and ## *p* < 0.01 derived from a paired *t*-test within groups, Weeks 0 vs. 12.

The results of the symptom questionnaire evaluation demonstrated that vaginal discharges and dysuria pain were significantly reduced in the MED-01-intake group after 6 and 12 weeks of intake compared with that at the baseline (*p* < 0.05), but there was no significant difference between the groups (Figure 3A,C). Moreover, the vaginal burning sensation decreased after ingestion of MED-01 and increased in the control group within the same period, but no statistically significant difference between the two groups was confirmed (Figure 3B). The vaginal odor decreased during the test period in both the groups; therefore, the difference between the two groups could not be confirmed (Figure 3D).

### 3.5. Effect of MED-01 on Vaginal Microbiota

**Figure 4:**
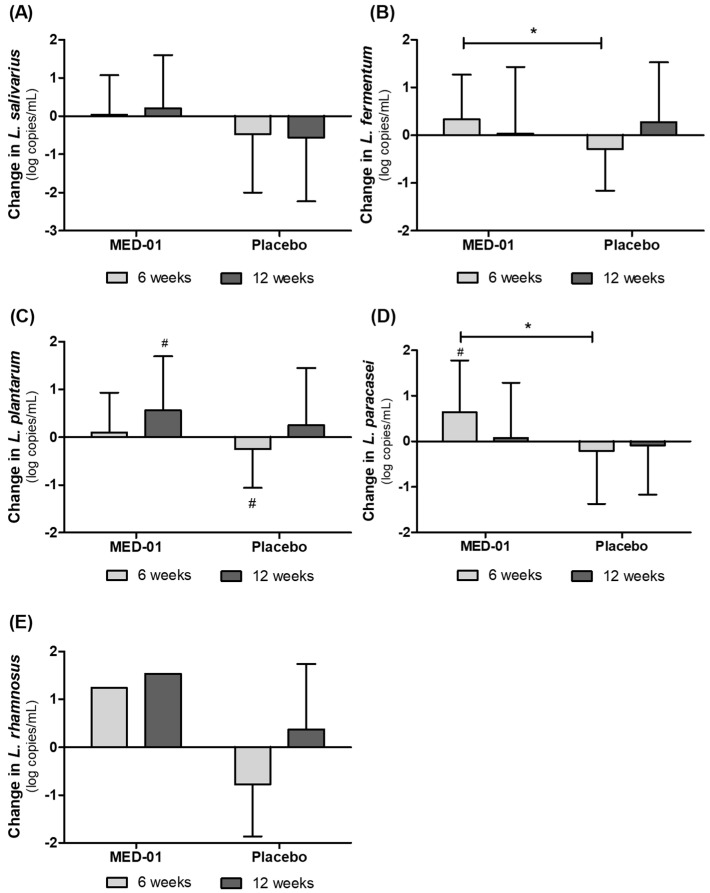
Effect of MED-01on vaginal lactic acid bacteria (LAB), as determined by quantitative PCR from baseline to 12 weeks after randomization of subjects. (**A**) *Ligilactobacillus salivarius*, (**B**) *Limosilactobacillus fermentum*, (**C**) *Lactiplantibacillus plantarum*, (**D**) *Lacticaseibacillus paracasei*, and (**E**) *Lacticaseibacillus rhamnosus*. Values are presented as the mean of change in log copies/mL ± SD. * *p* < 0.05 derived from a two-sample *t*-test between two groups, MED-01 vs. placebo; # *p* < 0.05 derived from a paired *t*-test within groups, Weeks 0 vs. 12.

**Figure 5:**
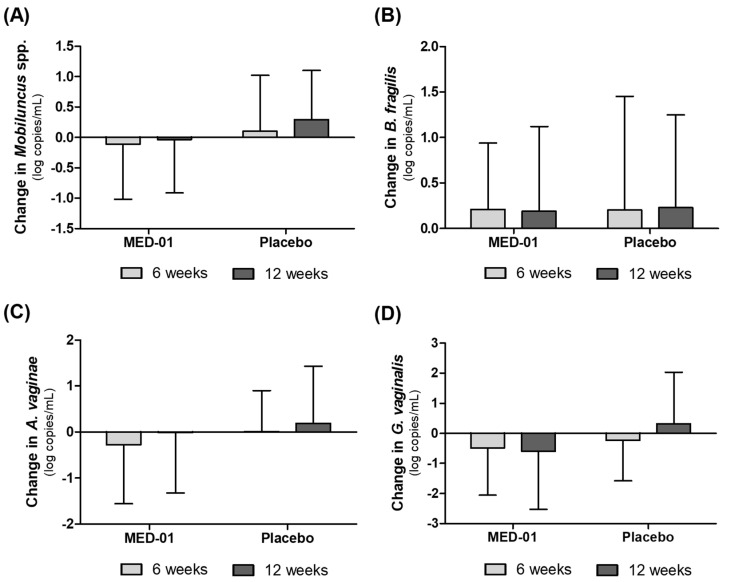
Effect of MED-01 on vaginal BV-inducing bacteria, as determined by quantitative PCR from baseline to 12 weeks after randomization of subjects. (**A**) *Mobiluncus* spp., (**B**) *Bacteroides fragilis,* (**C**) *Atopobium vaginae,* and (**D**) *Gardnerella vaginalis*. Values are presented as the mean of change in log copies/mL ± SD.

Changes in the vaginal microbiota after 12 weeks of MED-01 and placebo consumption were determined by qPCR analysis, and the results are shown in Figure 4 and Figure 5.

*L. fermentum* and *L. paracasei* significantly increased after 6 weeks of MED-01 intake compared with that after placebo intake (*p* < 0.05) (Figure 4B,D). There was no significant difference between the groups, but *L. plantarum* significantly increased in the MED-01-intake group after 12 weeks and significantly decreased in the placebo-intake group after 6 weeks compared with those at the baseline (Figure 4C).

In addition, after 12 weeks of MED-01 intake, vaginal *Mobiluncus* spp., *A. vaginae* and *G. vaginalis* decreased in the MED-01 group but increased in the control group. However, these differences were not significant (Figure 5A,C,D). *Bacteroides fragilis* increased in both groups without any significant difference (Figure 5B).

### 3.6. Safety of MED-01 and Placebo

A total of 101 randomized participants (50 in the MED-01 group and 51 in the placebo group) who consumed MED-01 or placebo capsules at least once were analyzed (Table 4). During the trial period, 16 AEs were reported in 12 subjects from the MED-01 group and 22 AEs, in 15 subjects from the placebo group. Six AEs, including gastrointestinal symptoms, such as gas/bloating, abdominal distension, nausea, constipation, and dyspepsia, were reported to be “possibly related” to MED-01 intake. These AEs occurred in 3 subjects each in the MED-01 or placebo groups. The AEs occurred at similar rates in the MED-01 and placebo groups, and the symptoms of AEs were also similar. In addition, of the excluded subjects, 6 subjects reported AEs, which were the above-mentioned gastrointestinal symptoms. Therefore, those AEs were considered to be due to maltodextrin used as a food supplement or to other factors unrelated to this study. No serious AEs were identified, and there was no statistically significant difference in the frequency and type of AEs that occurred during the study period between the two groups (*p* = 0.539).

The vital signs (pulse and blood pressure) and body weight of all subjects were found to be within the normal ranges in both the groups. In addition, there were no significant difference in inspection parameters, including hematology, blood chemistry, and urinalysis between the two groups after 12 weeks.

## 4. Discussion

Changes in the intestinal microbiota are closely related to the existing microbiota in the gut and ingested foods [25,26]. In addition, even if the same probiotic product is consumed, the efficacy and side effects differ depending on the individual [27]. Therefore, the valid results of clinical trials conducted abroad and the effect on Koreans may be different, suggesting the need to develop high-quality probiotics with functionality for Koreans. This clinical trial is the first to evaluate the effect of administrating a complex (MED-01) of five strains isolated from the vagina on the vaginal health of Korean women with pre-vaginitis symptoms.

The Nugent score is an index used to diagnose the degree of BV and has been consistently used in the field of BV research. Currently, PCR-based detection kits are also used, but the results obtained from these kits are difficult to compare with the research results from previous studies [28]. In addition, the Nugent score has a higher accuracy than Amsel criteria, which is another vaginitis diagnostic measure, and is therefore currently used as a new molecular diagnostics method for diagnosing BV [29]. *UREX*^TM^ probiotics or Respecta^®^ probiotics, which are known to be effective products for vaginal health, also decrease the Nugent score, confirming their efficacy in BV treatment [30]. Therefore, in the present study, the beneficial effect of probiotics on the subjects with BV was calculated using the Nugent score as the primary evaluation index. The Nugent score decreased in the MED-01-intake group and increased in the placebo-intake group, showing a statistically significant difference. Therefore, we confirmed that MED-01 was effective in alleviating BV. Moreover, MED-01 reduced dysuria, vaginal discharges, and vaginal burning sensations, which are symptoms of BV. Despite the increase in LAB in the vagina, the pH remained unchanged. Since a vaginal pH of 4.5 is generally regarded as normal, no pH shift is confirmed even by the growth of Lactobacilli. The same trend was reported by Russo et al. [27,31].

Recently, with the development of next-generation sequencing (NGS) technology, various studies have reported a correlation between vaginal infection and changes in vaginal microbiota. Studies on vaginal bacterial communities by NGS analysis have revealed two types of clusters dominated by Lactobacilli in BV-negative women and three types of clusters in BV-positive women. BV-positive subjects have a relatively high bacterial species diversity cluster profiles dominated by anaerobic species, including *G. vaginalis* [32,33]. Another study has reported similar results using qPCR; vaginal Lactobacilli decrease and anaerobic species such as *G. vaginalis* and *A. vaginae* increase in BV-infected patients [34]. In this study, the vaginal microbiota and the change in their numbers after ingestion of MED-01 and placebo were confirmed using qPCR analysis. The number of the 5 strains included in MED-01 increased in the vagina, indicating that MED-01 taken orally could reach the vagina. *L. fermentum* and *L. paracasei* significantly increased after 6 weeks of MED-01 intake compared with that after placebo intake and *L. plantarum* significantly increased in the MED-01-intake group after 12 weeks compared with baseline. This is consistent with the mechanism revealed in previous in vitro and in vivo experiments, indicating that ingested MED-01 improves the vaginal environment by migrating to the vagina and inhibiting pathogen adhesion and biofilm formation on the vaginal wall [17].

*Mobiluncus* spp., *G. vaginalis*, *A. vaginae*, and *B. fragilis* identified in this study are representative pathogens that increase during vaginitis [10]. *G. vaginalis* is the most representative indicator of vaginitis and is known to increase in the early stages of disease onset, exfoliate epithelial cells, and increase the inflammatory response [35]. *A. vaginae* forms a biofilm together with *G. vaginalis*, causing inflammation of epithelial cells and making it challenging to treat BV because of its antibiotic resistance [36]. *Mobiluncus* spp. highly influence the recurrence of BV and produce malic acid and trimethylamine, causing vaginal irritation and an unpleasant odor [37,38]. In a previous study, the administration of MED-01 to *G. vaginalis*-inoculated mice alleviated epithelial exfoliation by inhibiting the growth and adhesion of *G. vaginalis* in the vagina [17]. Vaginal epithelial exfoliation has been reported to be related to the Nugent score [39]. In this study, MED-01 intake reduced *Mobiluncus* spp. and *G. vaginalis* compared with placebo intake. Considering these results, it was confirmed that MED-01 ingestion alleviates BV by inhibiting the growth of pathogens in the vagina.

In the safety evaluation of MED-01 intake, there was no significant difference between the test groups in vital signs and urinalysis. In addition, the frequency of adverse and serious adverse events in clinical pathology tests, such as hematology and blood chemistry, did not differ between the test groups.

As this study is the first clinical trial test of MED-01, it was important to confirm whether the MED-01 strains were reached and settled in the vagina after ingestion. We plan to conduct a multivariate analysis of vaginal microbiota changes by MED-01 intake in further studies.

## 5. Conclusions

The present study demonstrated that MED-01 intake significantly reduces the Nugent score, a discriminant index of BV, and alleviates symptoms such as vaginal discharge, dysuria, and burning sensation, which may appear in BV without any adverse events. Additionally, it was confirmed that the intake of MED-01 increases the proportion of beneficial Lactobacilli and diminishes the harmful BV-causing pathogens through qPCR. However, there was no significant change in vaginal microbiota. Therefore, further studies on the changes in vaginal microbiota by ingestion of MED-01 are needed. Nevertheless, it was confirmed that the MED-01 intake group significantly decreased the Nugent score and alleviated the symptoms of BV compared to the control group. Therefore, we suggested the possibility of using MED-01 as a new and safe functional probiotic for women with symptoms of BV.

## Figures and Tables

**Table 1:** The Nugent scoring of Gram-stained smears for diagnosis of bacterial vaginosis.

Organism (Morphotype)	Number/Oil Immersion Field	Score
*Lactobacillus*-like (parallel sided, Gram-positive rods)	>30	0
	5–30	1
	1–4	2
	<1	3
	0	4
*Mobiluncus*-like (curved, Gram-negative rods)	>5	2
	<1–4	1
	0	0
*Gardnerella/bacteroides*-like (tiny, gram variable coccobacilli and pleomorphic rods with vacuoles)	>30	4
	5–30	3
	1–4	2
	<1	1
	0	0

The total Nugent score is calculated as the sum of the scores for each organism group. Total score: 0–3, Normal; 4–6, Intermediate; 7–10, Bacterial vaginosis.

**Table 2:** Demographic and lifestyle characteristics of the participants at the baseline.

Variable		MED-01 (*n* = 39)	Placebo (*n* = 37)	*p*-Value
Sex (female, %)		100	100	
Age (years)		39.56 ± 6.58	37.03 ± 7.15	0.111 ^T^
Drinking	(Y/N)	20/19	20/17	1.000 ^F^
Smoking	(Y/N)	4/35	1/36	0.163 ^F^
Frequency of exercising				
0–2 times/week		32 (82.05)	27 (72.97)	0.429 ^F^
3–7 times/week		7 (17.95)	10 (27.02)	
Frequency of showering				
1–3 times/week		4 (10.26)	3 (8.11)	0.668 ^F^
4–7 times/week		34 (87.18)	31 (83.78)	
2 or more times/week		1 (2.56)	3 (8.11)	
Usage of swimming pool	(Y/N)	1/38	0/37	1.000 ^F^
Usage of public baths	(Y/N)	1/38	3/34	0.418 ^F^
Usage of bidet	(Y/N)	11/28	6/37	0.210 ^C^
Types of sanitary products				
disposable sanitary pad	(Y/N)	36/3	34/3	1.000 ^F^
cotton sanitary napkins	(Y/N)	2/37	3/34	0.671 ^F^
menstrual cup	(Y/N)	2/37	1/36	1.000 ^F^
tampon	(Y/N)	3/36	7/30	0.186 ^F^
Frequency of intercourse				
none		12 (30.77)	13 (35.14)	0.251 ^F^
1 time/month		20 (51.28)	12 (32.43)	
1 time/week		4 (10.26)	9 (24.32)	
2–3 times/week		2 (5.13)	3 (8.11)	
4 or more times/week		1 (2.56)	0 (0.00)	

Values are presented as mean ± standard deviation (SD); percentages may not sum to 100 because of rounding; *p*-values were analyzed by ^C^ chi-square test, ^F^ Fisher’s exact test, or ^T^ two-sample *t*-test; Y/N: Yes/No.

**Table 3:** Number of participants with bacterial vaginosis (BV) status after consuming of food supplements as determined by the Nugent scores in the MED-01 and placebo groups.

Variable	MED-01 (*n* = 39)		Placebo (*n* = 37)		
	Baseline	12 Weeks	Baseline	12 Weeks	*p*-Value
Nugent score					
0–3 (Normal)	-	10 (25.6%)	-	5 (13.5%)	0.382
4–6 (Intermediate)	39 (100%)	23 (59.0%)	37 (100%)	24 (64.9%)	
7–9 (BV)	-	6 (15.4%)	-	8 (21.6%)	

Results are expressed as absolute numbers and percentages, respectively. For proportions, a chi-square two-tailed test was used.

**Table 4:** Safety assessment before and after 12 weeks of MED-01 and placebo intake.

Variable	MED-01 (*n* = 50)		Placebo (*n* = 51)		***p*-Value**
	Baseline	12 Weeks	Baseline	12 Weeks	
Hematology					
RBC (10^12^/L)	4.26 ± 0.29	4.30 ± 0.28	4.30 ± 0.31	4.25 ± 0.25	0.134 ^T^
Hb (g/dL)	12.64 ± 1.10	12.80 ± 1.09	12.83 ± 1.18	12.74 ± 1.32	0.075 ^W^
Hct (%)	38.15 ± 2.73	38.46 ± 2.63	38.66 ± 3.06	38.43 ± 3.30	0.229 ^T^
WBC (10^9^/L)	5.69 ± 1.54	5.83 ± 1.81	5.98 ± 1.75	5.89 ± 1.88	0.809 ^W^
Platelet (10^3^/µL)	242.92 ± 50.67	251.71 ± 54.35	269.63 ± 66.38	269.48 ± 62.27	0.215 ^T^
Neutrophil (%)	56.23 ± 7.44	56.35 ± 8.65	58.49 ± 9.86	56.54 ± 8.97	0.787 ^W^
Lymphocyte (%)	33.46 ± 6.89	33.52 ± 7.80	31.48 ± 8.69	32.84 ± 8.60	0.490 ^T^
Monocyte (%)	6.60 ± 1.81	6.45 ± 1.88	6.45 ± 2.09	6.75 ± 2.18	0.234 ^T^
Eosinophil (%)	2.40 ± 1.61	2.37 ± 1.66	2.24 ± 1.64	2.54 ± 2.20	0.204 ^W^
Basophil (%)	0.69 ± 0.34	0.59 ± 0.36	0.64 ± 0.31	0.58 ± 0.29	0.934 ^W^
MCV (fL)	89.74 ± 4.83	89.44 ± 4.95	90.02 ± 5.31	90.43 ± 6.19	0.819 ^T^
Blood chemistry					
AST (IU/L)	20.48 ± 8.54	20.56 ± 6.21	19.10 ± 4.98	19.28 ± 5.26	0.811 ^W^
ALT (IU/L)	15.48 ± 7.98	16.69 ± 9.01	14.76 ± 8.84	14.61 ± 9.29	0.630 ^W^
Protein (g/dL)	7.21 ± 0.34	7.23 ± 0.38	7.25 ± 0.35	7.25 ± 0.43	0.936 ^T^
Albumin (g/dL)	4.40 ± 0.26	4.43 ± 0.24	4.50 ± 0.27	4.43 ± 0.28	0.132 ^W^
Total bilirubin (mg/dL)	0.61 ± 0.26	0.64 ± 0.28	0.65 ± 0.31	0.60 ± 0.27	0.087 ^W^
ALP (IU/L)	54.62 ± 13.93	55.98 ± 12.73	53.27 ± 13.85	54.54 ± 13.17	0.371 ^T^
Creatinine (mg/dL)	0.67 ± 0.10	0.66 ± 0.10	0.64 ± 0.09	0.64 ± 0.09	0.678 ^T^
BUN (mg/dL)	12.40 ± 2.90	12.44 ± 2.77	11.16 ± 2.63	11.00 ± 2.54	0.991 ^W^
Uric acid (mg/dL)	4.20 ± 0.92	4.39 ± 1.20	4.25 ± 0.94	4.20 ± 1.00	0.169 ^W^
γ-GTP	15.32 ± 11.89	16.02 ± 10.56	17.37 ± 12.79	16.74 ± 10.95	0.767 ^W^
Anthropometry					
Systolic blood pressure (mmHg)	114.38 ± 13.73	118.47 ± 14.93	114.43 ± 12.73	113.35 ± 12.77	0.072 ^T^
Diastolic blood pressure (mmHg)	70.60 ± 11.29	71.36 ± 13.70	71.08 ± 8.79	70.11 ± 8.93	0.338 ^T^
Pulse (times/min)	78.10 ± 9.63	81.20 ± 10.83	81.96 ± 10.78	81.37 ± 9.03	0.112 ^T^
Body weight (kg)	58.08 ± 8.12	58.23 ± 8.49	57.20 ± 7.30	56.67 ± 7.20	0.605 ^T^

Safety assessments were performed on all participants in randomization (safety set, *n* = 101). Values are presented as mean ± SD. *p*-values were analyzed by ^T^ two-sample *t*-test or ^W^ Wilcoxon rank sum test between the groups. RBC, red blood cell; Hb, hemoglobin; Hct, hematocrit; WBC, white blood cell; MCV, mean corpuscular volume; AST, aspartate aminotransferase; ALT, alanine aminotransferase; ALP, alkaline phosphatase; BUN, blood urea nitrogen; γ-GTP, gamma-glutamic transpeptidase.

## Data Availability

The information about a clinical trial was registered at the Clinical Research Information Service, the Republic of Korea (accessed on 6 January 2023, https://cris.nih.go.kr/cris/search/detailSearch.do?search_lang=E&focus=reset_12&search_page=L&pageSize=10&page=undefined&seq=18347&status=5&seq_group=18347).

## Supplementary Materials

The following supporting information can be downloaded at: https://www.mdpi.com/article/10.3390/nu15020331/s1, Table S1: Primer sequence or kit information used for Quantitative Polymerase Chain Reaction (qPCR) [40,41,42,43,44].
