# Supplementary material for: Efficacy and Safety of MED-01 Probiotics on Vaginal Health: A 12-Week, Multicenter, Randomized, Double-Blind, Placebo-Controlled Clinical Trial
Source: Nutrients. 2023 Jan 9;15(2):331. doi: 10.3390/nu15020331 (PMC9863863; doi:10.3390/nu15020331)
Supplement: Supplementary file 1 [file nutrients-15-00331-s001.zip › nutrients-2086374-supplementary.pdf]

Table S1. Primer sequence or kit information used for Quantitative Polymerase Chain Reaction (qPCR)

Species		Primer sequence (5′ → 3′)	References
Mobiluncus spp.	F	CGC AAC CCT TGT CCT ATG TT	[1]
	R	GCG TGA AGC CCA AGA CAT AA	
A. vaginae	F	TAG GTC AGG AGT TAA ATC TG	[2]
	R	TCA TGG CCC AGA AGA CCG CC	
B. fragilis	F	GGG TAG GAG ACC CTT CTA TT	[2]
	R	CCA TGC CCG CTG ATT AC	
L. salivarius	F	GAC ATC CTT TGA CCA CCT AAG	[3]
	R	GGG ACT TAA CCC AAC ATC TC	
L. fermentum	F	GAG CGC AAC CCT TGT TAC TA	[4]
	R	GTG TGT AGC CCA GGT CAT AAG	
L. plantarum	F	CAA GCG TTG TCC GGA TTT ATT G	[4]
	R	CCA GTT TCC GAT GCA CTT CT	
L. paracasei	F	CAT GGG TAG CGA ACA GGA TTA G	[5]
	R	CCC AGG CGG AAT GCT TAA T	
L. rhamnosus	F	TGC TTG CAT CTT GAT TTA ATT TTG	[5]
	R	GGT TCT TGG ATT TAT GCG GTA TTA G	

* *A. vaginae, Atopobium vaginae; B. fragilis, Bacteroides fragilis; L. salivarius, Ligilactobacillus salivarius; L. fermentum, Limosilactobacillus fermentum; L. plantarum, Lactiplantibacillus plantarum; L. paracasei, Lacticaseibacillus paracasei; L. rhamnosus, Lacticaseibacillus rhamnosus.*

1. Oliveira, L.M.A.; Diniz, C.G.; Fernandes, A.A.S.; Souza-Sotte, D.M.K.; Freitas, M.C.R.; Machado, A.B.F.; Silva, V.L. Assessment of vaginal microbiota in Brazilian women with and without bacterial vaginosis and comparison with Nugent score. *The Journal of Infection in Developing Countries* **2018**, *12*, 127-136.
2. Malaguti, N.; Bahls, L.D.; Uchimura, N.S.; Gimenes, F.; Consolaro, M.E.L. Sensitive detection of thirteen bacterial vaginosis-associated agents using multiplex polymerase chain reaction. *BioMed research international* **2015**, *2015*.
3. Lai, C.-H.; Wu, S.-R.; Pang, J.-C.; Ramireddy, L.; Chiang, Y.-C.; Lin, C.-K.; Tsen, H.-Y. Designing primers and evaluation of the efficiency of propidium monoazide–Quantitative polymerase chain reaction for counting the viable cells of Lactobacillus gasseri and Lactobacillus salivarius. *journal of food and drug analysis* **2017**, *25*, 533-542.

4. Schwendimann, L.; Kauf, P.; Fieseler, L.; Gantenbein-Demarchi, C.; Schwenninger, S.M. Development of a quantitative PCR assay for rapid detection of Lactobacillus plantarum and Lactobacillus fermentum in cocoa bean fermentation. *Journal of microbiological methods* **2015**, *115*, 94-99.
5. Zhang, R.; Daroczy, K.; Xiao, B.; Yu, L.; Chen, R.; Liao, Q. Qualitative and semiquantitative analysis of Lactobacillus species in the vaginas of healthy fertile and postmenopausal Chinese women. *Journal of medical microbiology* **2012**, *61*, 729-739.
